# Association between Serum Soluble α-Klotho and Urinary Albumin Excretion in Middle-Aged and Older US Adults: NHANES 2007–2016

**DOI:** 10.3390/jcm12020637

**Published:** 2023-01-13

**Authors:** Kaixi Chang, Yupei Li, Zheng Qin, Zhuyun Zhang, Liya Wang, Qinbo Yang, Baihai Su

**Affiliations:** 1 Department of Nephrology, West China Hospital, Sichuan University, Chengdu 610041, China; 2 Med+ Biomaterial Institute of West China Hospital, West China School of Medicine of Sichuan University, Chengdu 610041, China; 3 Med-X Center for Materials, Sichuan University, Chengdu 610041, China

**Keywords:** Klotho, albuminuria, albumin-to-creatinine ratio, chronic kidney disease, biomarker

## Abstract

(1) Background: Preclinical and clinical studies on the anti-aging effect of α-Klotho are emerging. Urinary albumin excretion (UAE) is a well-known biomarker of kidney injury and generalized damage in the cardiovascular system. However, the potential relationship between α-Klotho and UAE is limited and controversial. This study aimed to quantify this relationship in the general middle-aged and elderly population from the National Health and Nutrition Survey (NHANES) 2007–2016. (2) Methods: Serum α-Klotho was measured by enzyme-linked immunosorbent assay. UAE was assessed by the albumin-to-creatinine ratio (ACR). After adjusting for several confounding variables, the relationship between α-Klotho and ACR was analyzed by weighted multivariable logistic regression, subgroup analysis, and interaction tests. A generalized additive model (GAM) with smooth functions using the two-piecewise linear regression model was used to examine the potential nonlinear relationship between α-Klotho and ACR. (3) Results: Among 13,584 participants aged 40–79 years, we observed an independent and significant negative correlation between α-Klotho and ACR (β = −12.22; 95% CI, −23.91, −0.53, *p* = 0.0448) by multivariable logistic regression analysis, especially in those with age ≥ 60 years, pulse pressure (PP) ≥ 60 mmHg, hypertension or diabetes. We further discovered the nonlinear relationship between α-Klotho and ACR by GAM, revealing the first negative and then positive correlations with an inflection point of 9.91 pg/mL between α-Klotho and ACR. (4) Conclusions: A dose-response relationship between α-Klotho and ACR was demonstrated, and the negative correlation therein indicated that α-Klotho has potential as a serum marker and prophylactic or therapeutic agent despite its metabolic and effective mechanisms needing to be further explored.

## 1. Introduction

The α-Klotho gene is an aging suppressor gene highly expressed in the kidney, mainly in the renal tubules [1], and serves as the obligate co-receptor for fibroblast growth factor 23 (FGF23) [2]. FGF23-knockout mice contributed greatly to the initial discovery of α-Klotho [3], and two paralogous genes were subsequently discovered and termed β-Klotho [4] and γ-Klotho [5]. α-Klotho is classified as a trans-membrane and soluble circulating form [6], which has gained attention due to its pleiotropic effect in the pathophysiology of multiple diseases, such as chronic kidney disease (CKD) [7], diabetes [8], cardiovascular diseases [9], and cancer [10]. It was reported that low serum klotho concentration (<666 pg/mL) was associated with a 31% higher risk of all-cause mortality (compared to klotho concentration > 985 pg/mL) [11]. Considerable data also suggest that α-Klotho is not only a biomarker of healthy aging [12] but also a valuable therapeutic candidate target in aging-related disorders due to its anti-aging properties [13]. Unfortunately, the interconnectedness of the involved age-related diseases is often ignored in current clinical practice while comorbidity tends to increase with aging. Various animal models indicated that α-Klotho could be used in gene therapy as a longevity-associated gene [14], and the restoration of α-Klotho in deficient states could reverse the premature aging-related phenotype [15]. In this regard, measuring the expression status or content of α-Klotho in vivo as a biological marker will contribute to exploring its potential causative and compensatory mechanisms [12,16], leading to its early clinical detection and successful intervention.

CKD is defined as abnormalities of the kidney structure or function present for >3 months, with implications for health. By the end of this century, CKD is expected to become the second leading cause of death in countries with long life expectancy due to the progressive aging of the world population [17], which is associated with high prevalence, poor outcome, and high costs [18]. Notably, α-Klotho can induce phenotypes prone to the development of CKD and serve as a useful clinical biomarker for predicting the progression of CKD. A post hoc analysis of a prospective cohort study suggested that the risk of reaching the primary composite outcome of the progression of CKD and death was significantly higher in patients with α-Klotho levels ≤ 396.3 pg/mL than in patients with α-Klotho levels > 396.3 pg/mL (hazard ratio, 2.03; 95% confidence interval, 1.07–3.85; *p* = 0.03) [19]. Specifically, soluble Klotho was reported to start to decline in the early stage of CKD [20,21]. Additionally, there are reviews suggesting that Klotho deficiency can also be a causative factor in the progression of kidney damage [22,23]. Klotho supplementation might be a wise move by preserving remnant kidney function as well as reducing CKD consequences through improving Pi metabolism and inhibiting oxidative, apoptosis, and senescence [24].

Albuminuria, also known as increased urinary albumin excretion (UAE), is generally defined as an albumin-to-creatinine ratio (ACR) ≥ 30 mg/g [25,26,27]. Albuminuria can be the only characteristic of early-stage CKD patients with normal eGFR and is associated with adverse health outcomes [28]. Moreover, even individuals with an ACR of 10–29 mg/g who have normal eGFR already have a 50% higher risk of all-cause death in the general population [25]. More importantly, growing evidence suggests that albuminuria is also a sign of altered glomerular hemodynamics, abnormal tubular function, activation of the renin-angiotensin system, and generalized damage and systemic inflammation in the cardiovascular system [29]. Currently, with increasingly gaining attention, ACR is suggested to be incorporated into the risk stratification management of cardiovascular diseases and CKD [30,31,32].

Drew et al. reported that a higher soluble Klotho level was independently associated with a lower risk of decline in kidney function (defined as eGFR decline) without albuminuria tested in a follow-up [33]. And a recent study by Zhang et al. found that α-Klotho levels were negatively associated with the prevalence of CKD (as a dependent variable), and albuminuria (as a categorical dependent variable) was the evidence of kidney damage together to define CKD [34]. However, these previous publications did not focus on the association of α-Klotho and ACR in the general population. Therefore, this study aims to explore the relationship between α-Klotho and ACR in the US general population, thus providing more information and evidence to facilitate improved research guidance regarding α-Klotho for kidney health and healthy aging.

## 2. Materials and Methods

### 2.1. Sample Population

The National Health and Nutrition Examination Survey (NHANES) is an ongoing project using a complex, multistage, probability-sampling design aiming to assess the health and nutritional status of the US noninstitutionalized population, which was approved by the National Center for Health Statistics Institutional Review Board, and all participants gave informed consent. It collects questionnaire data through interviews, performs health screenings at a mobile examination center (MEC), and collects specimens for laboratory testing. More details about NHANES can be found on its website (https://www.cdc.gov/nchs/nhanes/about_nhanes.htm, accessed on 6 January 2023 [35].

**Figure 1:**
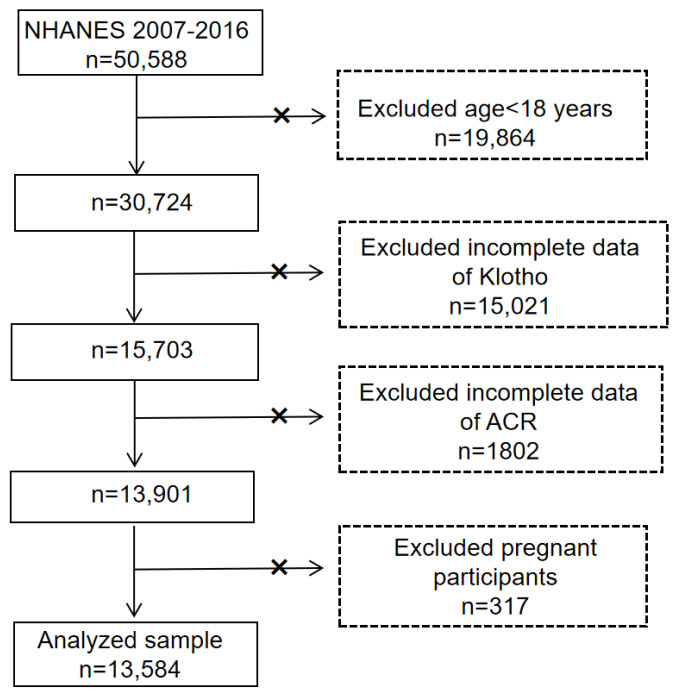
Flowchart of the participants’ selection from NHANES 2007–2016. Abbreviations: ACR = albumin–to–creatinine ratio.

Our study combined five NHANES cycles (2007–2008, 2009–2010, 2011–2012, 2013–2014, and 2015–2016) containing the complete set of variables, with 50,588 participants in total. We excluded those aged < 18 years (n = 19,864), missing klotho (n = 15,021), missing ACR (n = 1802), and pregnant (n = 317). Ultimately, 13,584 participants were included in the final analyses (Figure 1).

During the five cycles of the NHANES project, blood samples were obtained from participants aged 40–79 years, stored in dry ice at −80 °C at the Centers for Disease Control and Prevention (CDC) in Atlanta, Georgia, and delivered to the Northwest Lipid Metabolism and Diabetes Research Laboratory at the University of Washington in Seattle, Washington. Serum Klotho levels in the fresh-frozen samples were measured using the Human Soluble α-Klotho Assay Kit (Immuno-Biological Laboratories Co., Ltd., Fujioka, Japan; intra-assay coefficient of variation: <3.6%, interassay coefficient of variation: <11.4%, assay sensitivity: 6 pg/mL). Every sample was tested twice, with the average of the results serving as the final value. Analytical results are automatically transferred from the instrument to the laboratory Oracle management system for evaluation by the regional supervisor. Reproducible analyses are those that provide repeatable results of 10% or more for the samples. The analysis was invalidated and the sample analysis was rerun if the value of the quality control sample was not within 2 standard deviations (SDs) of the target value. In our analysis, α-Klotho was designed as an independent variable.

### 2.2. Assessment of Increased UAE

Urine samples of NHANES participants were obtained in MEC. Urinary albumin and creatinine were determined using a single spot urine sample by solid-phase fluorescence immunoassay and a modified Jaffe kinetic method. ACR was calculated based on urinary albumin (mg) concentration divided by urinary creatinine (g) concentration. Microalbuminuria and macroalbuminuria refer to spot urine ACR values of 30 to <300 mg/g and >300 mg/g, respectively [35]. ACR was treated as a continuous outcome variable in statistical analysis.

### 2.3. Covariates

The confounders used in the models fell into two categories, which could theoretically affect both main variables and be compatible with statistical principles (i.e., statistically relevant or have an effect on the coefficient of more than 10%).

Sociodemographic variables were self-reported via computer-assisted questionnaires, including age (year), gender (male or female), race (Mexican American, other Hispanic, non-Hispanic White, non-Hispanic Black, other races), and education level (less than high school, high school or General Equivalent Diploma (GED), above high school). Health conditions included the following items: Body mass index (BMI) was calculated as weight in kilograms divided by height in meters squared. We classified BMI as normal <25, overweight 25–30, and obese >30 kg/m [2] when it was treated as a categorical variable [36]. High-density lipoprotein cholesterol (HDL-C) and triglycerides were also used to indicate metabolic status [37]. Alanine aminotransferase (ALT) and aspartate aminotransferase (AST) were included because hepatic histological lesions are significantly associated with abnormal albuminuria [38]. HDL-C, triglycerides, ALT, and AST were measured enzymatically with a Hitachi-704 Analyzer (Roche Diagnostics, Indianapolis, IN, USA). Diabetes (yes or no) was defined as the treatment or medical diagnosis of hypoglycemia with a hemoglobin A1c ≥ 6.5%, a fasting blood glucose ≥ 126 mg/dL, or a 2-h blood glucose ≥ 200 mg/dL [39]. Systolic and diastolic blood pressure were measured by trained and certified clinical staff from the right arm of the participant in a seated position, using a conventional mercury sphygmomanometer, and pulse pressure (PP) was then obtained from systolic pressure minus diastolic pressure [40]. Hypertension (yes or no) was defined as the use of antihypertensive medications, a medical diagnosis of hypertension, or three consecutive measurements of systolic blood pressure at ≥140 mmHg or diastolic blood pressure at ≥90 mmHg [41]. All detailed measurement procedures for all of these variables are posted at www.cdc.gov/nchs/nhanes/, accessed on 6 January 2023 [42].

### 2.4. Statistical Analysis

All statistical analyses were performed in accordance with the CDC guidelines. Considering that NHANES employs a complex probability sample design and oversamples some population groups to ensure adequate representation, sample weights were utilized to combine the survey cycles and estimate the mean values and standard errors. Continuous variables are represented by the mean plus standard deviation (SD) or median (interquartile range, IQR), and categorical parameters are expressed as proportions. α-Klotho levels were log base 2 transformed due to its right-skewed distribution after the normality test, and it is also rational to explain that the change of the dependent variable is caused by per doubling of the exposure.

To determine differences in descriptive analyses, statistical analysis was performed on the student’s weighted *t*-test (for continuous variables) or weighted chi-square test (for categorical variables). Multivariable logistic regression was performed to examine the association between α-Klotho levels as the dependent variable and ACR as the independent variable in three different models to provide statistical inference. There was no covariate adjustment in model 1. Age, gender, and race were modified in model 2. Model 3 was adjusted for age, gender, race, education level, BMI, HDL-C, triglycerides, ALT, AST, diabetes, hypertension, and PP. In sensitivity analysis, α-Klotho was converted from a continuous variable to a categorical variable (tertiles) to evaluate the results’ robustness, and the UAE among the tertiles of these parameters was tested by linear test for trend. A generalized additive model (GAM) with smooth functions was used to further evaluate the potential nonlinear relationships between α-Klotho and ACR. When nonlinearity was found, we further determined the inflection point using a recursive algorithm and constructed a weighted two-piecewise linear regression model.

Subgroup analysis of the association of the α-Klotho level with ACR was conducted with stratified factors including gender (male/female), age (40–59/≥60 years), BMI (normal weight/overweight/obesity), hypertension (yes/no), diabetes (yes/no), PP (<60/≥60 mmHg), and race. These stratified factors were also handled as predetermined possible effect modifiers.

All analyses were performed using R version 3.4.3 (http://www.R-project.org, accessed on 6 January 2023, The R Foundation) and Empower software (www.empowerstats.com, accessed on 6 January 2023; X&Y Solutions, Inc., Boston, MA, USA). The statistical significance level was set as a two-sided *p*-Value of <0.05.

## 3. Results

### 3.1. Baseline Features

The clinical and biochemical characteristics of participants according to the α-Klotho tertiles are shown in Table 1. A total of 13,584 participants selected from NHANES 2007–2016 were involved in our study. Among them, 48.43% were males, with an average age of 57.65 ± 10.83 years. The median (IQR) of serum Klotho was 803.10 (655.50–994.23) pg/mL. The prevalence of albuminuria was 13.99% (microalbuminuria, 11.35%; macroalbuminuria, 2.64%).

Among the three α-Klotho tertiles, differences with statistical significance were observed in age, gender, race, education level, BMI, HDL-C, triglycerides, ALT, AST, diabetes, hypertension, PP, and urine albumin (all *p* < 0.05). Participants with increased Klotho level were likely to be 40–59 years old, female, and had lower PP in our study.

### 3.2. The Association between α-Klotho and ACR

Our results suggested that higher α-Klotho was associated with decreased ACR, as shown in Table 2. This association was significant both in the crude model (β = −18.07; 95% CI, −32.95, −3.19, *p* = 0.0197) and the minimally adjusted model 1 (β = −3.24; 95% CI, −6.39, −0.09, *p* = 0.0476). In the fully adjusted model 2, the negative association between α-Klotho and ACR remained stable (β = −12.22; 95% CI, −23.91, −0.53, *p* = 0.0448), indicating that each 2-fold increase in α-Klotho was associated with 12.22 mg/g of decreased ACR.

The negative correlation was still significant despite α-Klotho being in either linear or categoric analyses. In fully adjusted model 2, the highest tertile of α-Klotho (≥918.90 pg/mL) significantly correlated to a lower ACR (β = −12.67; 95% CI, −22.86, −2.47, *p* = 0.0179) compared with the lowest tertile of α-Klotho (<704.00 pg/mL). The *p*-Values for trend were 0.0041, 0.0294, and 0.0176 for the crude model, model 1, and model 2, respectively (Table 2). In the fully adjusted model, the results of multivariate logistic regression are shown in Table 3. Female, non-Hispanic, white individuals had lower ACR, whereas ACR was higher in those with abnormal BMI, elevated triglycerides and PP, diabetes, and hypertension.

Furthermore, the potential nonlinear relationship between α-Klotho and ACR was revealed by GAM with smooth curve fittings, and the two-piecewise linear regression model displayed the inflection point of 9.91 pg/mL using the nonlinear model (log-likelihood ratio < 0.001, Table 4). As the α-Klotho level was doubled, the level of ACR first decreased by −39.95 mg/g (95% CI, −58.82, −21.07, *p* < 0.0001) and then increased by 41.02 mg/g (95% CI, 8.47, 73.57, *p* = 0.0135).

### 3.3. Subgroup Analysis

**Figure 2:**
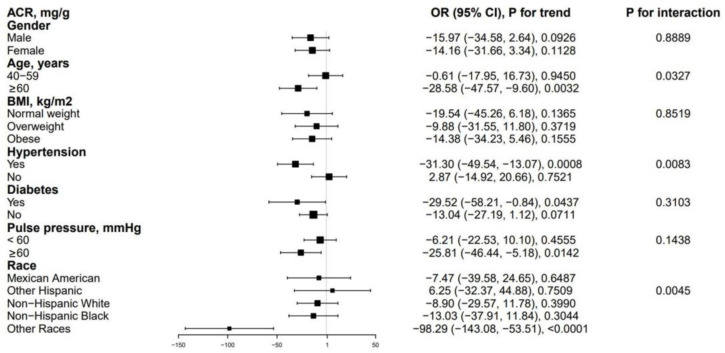
Subgroup analysis for the association between α-Klotho and ACR. Abbreviations: ACR = albumin–to–creatinine ratio, BMI = body mass index.

In the subgroup analysis (Figure 2), we found a significantly negative correlation between α-Klotho and ACR in those with age ≥ 60 years, PP ≥ 60 mmHg, hypertension, or diabetes. Additionally, age, hypertension, and race significantly affected the negative association between α-Klotho and ACR (*p*-interactions as 0.0327, 0.0083, 0.0045, respectively).

## 4. Discussion

To the best of our knowledge, this is the first study to investigate the relationship between the novel marker α-Klotho and ACR in a nationally representative cohort in the general US population. Baseline characteristics of the study population indicated that participants with higher Klotho levels were likely to be younger (40–59 years old), female, and, in our study, had lower PP, which was attributed to a possible negative correlation of α-Klotho with age and pulse pressure, and its potential correlation with sexual steroid hormones [13,33,43,44,45]. We first assumed a linear association and confirmed a robust negative independent correlation between α-Klotho and ACR in models adjusted for covariates and in subgroup analyses. This correlation was more significant in those with age ≥ 60 years, PP ≥ 60 mmHg, hypertension, or diabetes. In addition, we found that α-Klotho and ACR were not simply incremental relationships via a trend test. A further GAM with smooth curve fittings using the two-piecewise linear regression model was applied to obtain the inflection point of 9.91 pg/mL, proving the first negative and then positive correlation between them with statistical significance.

Correspondingly, our findings of a negative correlation between α-Klotho and ACR were in line with some human studies [8,24,46]. Microalbuminuria was proven to be associated with serum soluble Klotho deficiency in patients with type 1 diabetes in a cross-sectional single-center study [46]. Decreased urinary Klotho was also observed even in individuals with preserved GFR and pathological findings of albuminuria alone [24], and vice versa. The antialbuminuric effect was associated with increased serum and urinary Klotho levels [47]. However, these studies were limited by specific patient populations (patients with diabetes or CKD), small sample sizes (N < 100), and did not comprehensively depict the relationship between α-Klotho and albuminuria. Our study found that ACR decreased with α-Klotho in the range of <9.91 pg/mL, and above this value, ACR increased with increasing α-Klotho. In fact, the inflection point of 9.91 pg/mL we observed is of critical significance. On the one hand, the specific numerical changes in ACR treated as a continuous indicator can more fully reflect its risk threshold. On the other hand, this dose-response relationship reminded us to pay more attention to the management of α-Klotho in future clinical practice by maintaining its level in a relatively appropriate range, especially when its mechanism is not sufficiently clarified. The reality is that studies on biosynthesis and metabolism of α-Klotho are scarce, and only by correctly understanding the pathway of its development in vivo can it provide a basis for further potential application as a prophylactic or therapeutic agent. It was also reported that CKD is a state of Klotho deficiency in the kidney, plasma, and urine [24], while no association was found between serum and urinary Klotho, indicating that urinary Klotho is not the result of glomerular filtration [47]. Hu et al. also reported that soluble Klotho is not filtered across the glomerular barrier and that urinary Klotho originates in the renal tubules [48]. Interestingly, albuminuria may directly decrease Klotho expression through epigenetic mechanisms or through the recruitment of the inflammatory response [28]. In summary, the complex pathophysiologic mechanisms suggested that Klotho downregulation is not merely an early biomarker for kidney damage, but also one of the principal complications of CKD and may play a pathogenic role in CKD progression [24]. Additionally, due to the complexity of the mechanism, whether serum Klotho reflects kidney function remains controversial in some studies [49,50], which requires high-quality research evidence.

Furthermore, the bidirectional effects of α-Klotho and urinary albumin were found in various animal models, and global overexpression or exogenous supplementation with Klotho resulted in a reduction in UAE [51]. In contrast, mice with a genetic propensity for albuminuria have increased urinary Klotho excretion [52]. The potential mechanisms of such an interaction may involve suppressing the induction of proinflammatory cytokines, protecting against podocyte injury directly in situ or by autocrine or paracrine effects [52], limiting nucleotide-binding oligomerization domain-like pyrin domain containing protein 3 inflammasome-mediated pyroptosis and promoting autophagy [53], influencing sodium/phosphate cotransporter activity and oxidative stress, further regulating vascular calcification and endothelial dysfunction [54,55]. These mechanisms can, in turn, crosstalk with each other. Hence, our study provided reliable information for further studies, which are expected to determine the underlying mechanisms of this dose-response relationship between α-Klotho and ACR and to identify effective therapeutic windows for α-Klotho administration.

The large sample size of this study allowed us to conduct subgroup analyses to identify selected populations that are most likely to benefit from Klotho management or modulation. We found that age strongly modified the negative association between α-Klotho and ACR, and this negative association was more pronounced in those aged ≥ 60 years than in those aged 40–59 years. This concurs with other findings indicating weaker effects of Klotho on relatively young individuals [56,57,58]. It is undeniable that most studies have focused on mid- to end-of-life applications, and data from early-life interventions are sparse and require a more robust evaluation. Additionally, we found that elevated PP and the presence of hypertension or diabetes were potential effect modifiers as well, possibly because these states inherently accelerate senescence [59,60]. The enhanced senescence of renal tubules and vascular cells leads to endothelial dysfunction and impaired vasculogenesis [61], suggesting the presence of complex physiologically relevant feedback loops. It cannot be excluded that genetic polymorphisms of the Klotho gene also contribute to such physical states [62,63], even in relatively young and healthy individuals [58]. Gender and BMI were not significant modifiers in our study, which has also been demonstrated in several human studies [8,64], as well as animal studies [65]. Further large-scale prospective studies are required to verify our findings in different groups of healthy individuals. In addition, our study has several other advantages. The large sample size guarantees a high statistical power, as the representative of the entire U.S. population guarantees a high degree of external validity, and all variables are collected in a standardized and homogenous way.

We also acknowledge that our study has some potential limitations. Similar to any epidemiological investigation, unmeasured confounding variables may affect the correlation between α-Klotho and ACR. For example, serum FGF23, a central hormone to regulate phosphate homeostasis, is currently regarded as a potential marker of kidney function (both eGFR and tubular function) [66,67]. Considering the uncertainty of whether FGF23 has Klotho-dependent traditional and on-target effects or not, Klotho levels were influenced by FGF23 according to different study designs [68,69]. Therefore, further multicenter cohort studies remain to be carried out to clarify this point by incorporating more potential confounders. Due to the incompleteness of variable data in the NHANES database, along with trying to avoid the influence of too subjective indicators on the results, the selection of covariates was primarily based on widely prevalent health conditions and risk factors reported in the previous literature, which was described in the Section 2.3. For the purpose of performing sensitivity analysis and making the results more robust and reliable, our artificial categorization of continuously distributed variables has some inherent disadvantages, such as a loss of power, the possibility of inaccurate estimation, and difficulty comparing results across studies. Together with the limitation inherent to cross-sectional studies, our results can only provide clues to the causality of α-Klotho and ACR but cannot show the exact causality, thus, future evaluation in realistic clinical practice or longitudinal studies is still needed. Additionally, the interpretation of the results of the present study needs to take regional and ethnic differences and age groups (other than 40–79 years old) into consideration.

## 5. Conclusions

In conclusion, our study found an independent negative correlation and a potential dose-response relationship between α-Klotho and ACR, which may shed light on the uncertainly understood independent link between α-Klotho and ACR in the general population and has potential translational implications for identifying potential target population groups to reduce albuminuria.

## Figures and Tables

**Table 1:** Baseline characteristics of the study population according to α-Klotho tertiles (n = 13,584).

Characteristic	α-Klotho Levels Tertiles, pg/mL				*p*-Value for Trend
	Overall	<704.00	704.00–918.90	≥918.90	
No. of participants	13,584	4528	4526	4530	
α-Klotho (pg/mL), median (IQR)	803.10 (655.50–994.23)	594.10 (520.58–655.50)	803.05 (752.40–856.30)	1100.20 (994.20–1269.85)	<0.001
Age (year), mean (SD)	57.65 ± 10.83	58.86 ± 11.01	57.49 ± 10.77	56.59 ± 10.59	<0.001
40–59 years, %	7415, 54.59%	49.71	55.52	58.52	
60–79 years, %	6169, 45.41%	50.29	44.48	41.48	
Gender, %					<0.001
Male	48.43	51.46	49.60	44.24	
Female	51.57	48.54	50.40	55.76	
Race, %					<0.001
Mexican American	15.92	15.90	16.59	15.25	
Other Hispanic	11.52	10.29	11.73	12.54	
Non-Hispanic White	42.99	45.85	44.56	38.57	
Non-Hispanic Black	19.71	19.21	16.28	23.62	
Other Races	9.86	8.75	10.83	10.02	
Education level, %					<0.001
below high school	28.14	29.09	27.57	27.75	
High school or GED	22.11	23.37	22.29	20.68	
Above high school	49.75	47.55	50.13	51.57	
BMI (kg/m^2^), mean (SD)	29.71 ± 6.66	29.81 ± 6.38	29.75 ± 6.64	29.58 ± 6.94	0.006
Normal weight <25, %	23.88	22.11	23.49	26.02	
Overweight 25–29.9, %	34.66	35.83	34.80	33.36	
Obese ≥ 30, %	41.46	42.06	41.71	40.62	
HDL-C (mmol/L)	1.37 ± 0.43	1.37 ± 0.44	1.36 ± 0.42	1.39 ± 0.43	<0.001
Triglycerides (mmol/L)	1.90 ± 1.60	1.99 ± 1.92	1.89 ± 1.38	1.81 ± 1.46	<0.001
ALT (IU/L)	25.56 ± 18.99	24.68 ± 15.60	25.06 ± 19.58	26.94 ± 21.28	<0.001
AST (IU/L)	26.42 ± 16.68	25.62 ± 13.71	25.66 ± 13.74	27.98 ± 21.30	<0.001
Diabetes, %					0.018
Yes	17.71	18.73	16.48	17.92	
no	82.29	81.27	83.52	82.08	
Hypertension, %					<0.001
Yes	46.25	49.76	43.70	45.30	
no	53.75	50.24	56.30	54.70	
Pulse pressure, %					<0.001
<60	63.86	60.04	65.21	66.32	
≥60	36.14	39.96	34.79	33.68	
Urine albumin, %					<0.001
Normal (<30)	86.01	83.92	87.27	86.82	
Microalbuminuria (30–299)	11.35	12.52	10.41	11.13	
Macroalbuminuria (≥300)	2.64	3.56	2.32	2.05	

Abbreviations: GED = general educational development; BMI = body mass index; HDL-C = high-density lipoprotein-cholesterol; ALT = alanine transaminase; AST = aspartate transaminase. Note: Mean (SD) and median (IQR) for continuous variables: the *p*-Value was calculated by weighted linear regression. Percent for categorical variables: the *p*-Value was calculated by weighted chi-square test.

**Table 2:** Association between log2 α-Klotho and ACR.

Outcome	β (95%CI) ^1^, *p*-Value		
	Crude Model ^2^	Model 1 ^3^	Model 2 ^4^
α-Klotho (continuous)	−18.07 (−32.95, −3.19), 0.0197	−3.24 (−6.39, −0.09), 0.0476	−12.22 (−23.91, −0.53), 0.0448
α-Klotho (tertiles)			
T1	Reference	Reference	Reference
T2	−20.26 (−32.85, −7.67), 0.0023	−3.04 (−5.93, −0.16), 0.0422	−13.92 (−24.68, −3.16), 0.0139
T3	−19.31 (−32.19, −6.44), 0.0043	−3.34 (−6.30, −0.38), 0.0300	−12.67 (−22.86, −2.47), 0.0179
*p* for trend	0.0041	0.0294	0.0176

^1^ 95% Cl: 95% confidence interval. ^2^ Crude Model: no covariates were adjusted. ^3^ Model 1: adjusted for age, gender, and race. ^4^ Model 2: adjusted for age, gender, race, education level, BMI, HDL-C, triglycerides, ALT, AST, diabetes, hypertension, and pulse pressure.

**Table 3:** Multivariate logistic regression models of ACR.

Variables	β (95%CI)	*p*-Value
Log 2 α-Klotho		
per one-unit increase	−12.22 (−23.91, −0.53)	0.0448
Age (year)	−0.53 (−1.04, −0.01)	0.0503
Female (versus male)	−13.37 (−25.51, −1.23)	0.0348
Race (versus Mexican American)		
Other Hispanic	3.40 (−32.63, 39.44)	0.8538
Non-Hispanic white	−26.45 (−47.52, −5.39)	0.0168
Non-Hispanic black	−17.47 (−38.89, 3.95)	0.1151
Other race/ethnicity	4.93 (−36.32, 46.19)	0.8155
Education level (versus less than high school)		
High school or GED	−0.68 (−17.15, 15.80)	0.9363
Above high school	−7.03 (−20.69, 6.62)	0.3170
BMI (kg/m^2^) (versus normal weight < 25)		
Overweight 25–29.9	−10.68 (−17.04, −4.31)	0.0018
Obese ≥ 30	−3.85 (−0.68, −14.99)	0.5014
HDL-C (mmol/L)	18.58 (−0.26, 37.41)	0.0579
Triglycerides (mmol/L)	7.86 (1.04, 14.67)	0.0277
ALT (IU/L)	−0.58 (−0.99, −0.17)	0.0069
AST (IU/L)	0.34 (−0.10, 0.77)	0.1360
Diabetes (no versus yes)	−85.73 (−114.11, −57.35)	<0.0001
Hypertension (no versus yes)	−21.24 (−29.30, −13.18)	<0.0001
Pulse pressure (elevated versus normal)	34.65 (21.78, 47.53)	<0.0001

Note: Units for continuous variables and reference groups for categorical variables are presented alongside the variables. The β of ACR was each unit increase of continuous variables and compared with the reference group for categorical variables.

**Table 4:** Threshold effect analysis of α-Klotho on ACR using the two-piecewise linear regression model.

Models	ACR	
	β (95%CI)	*p*-Value
Model I		
One line slope	−14.31 (−27.13, −1.50)	0.0286
Model II		
Turning point (K)	9.91	
<9.91 slope 1	−39.95 (−58.82, −21.07)	<0.0001
>9.91 slope 2	41.02 (8.47, 73.57)	0.0135
slope 2-slope 1	80.97 (37.18, 124.76)	0.0003
Predicted at 9.91	28.15 (17.98, 38.33)	
Log likelihood ratio test	<0.001	

Model I: linear analysis. Model II: nonlinear analysis Log-likelihood ratio test: A *p*-Value <0.05 means Model II is significantly different from Model I, which indicates a nonlinear relationship. Abbreviations: ACR = albumin–to–creatinine ratio.

## Data Availability

The data for this study can be found in www.cdc.gov/nchs/nhanes/, accessed on 6 January 2023.
